# Optimized Biocatalytic Synthesis of 2‐Selenopyrimidine Nucleosides by Transglycosylation**

**DOI:** 10.1002/cbic.202100067

**Published:** 2021-03-31

**Authors:** Katja F. Hellendahl, Felix Kaspar, Xinrui Zhou, Zhaoyi Yang, Zhen Huang, Peter Neubauer, Anke Kurreck

**Affiliations:** ^1^ Technische Universität Berlin Faculty III Process Sciences, Institute of Biotechnology Chair of Bioprocess Engineering Ackerstraße 76 13355 Berlin Germany; ^2^ BioNukleo GmbH Ackerstraße 76 13355 Berlin Germany; ^3^ Sichuan University, College of Life Sciences Key Laboratory of Bio-Resource and Eco-Environment Ministry of Education No. 17 People's South Road Section 3 610041 Chengdu P. R. China

**Keywords:** nucleosides, selenium, transglycosylation, equilibrium constant, nucleoside phosphorylase

## Abstract

Selenium‐modified nucleosides are powerful tools to study the structure and function of nucleic acids and their protein interactions. The widespread application of 2‐selenopyrimidine nucleosides is currently limited by low yields in established synthetic routes. Herein, we describe the optimization of the synthesis of 2‐Se‐uridine and 2‐Se‐thymidine derivatives by thermostable nucleoside phosphorylases in transglycosylation reactions using natural uridine or thymidine as sugar donors. Reactions were performed at 60 or 80 °C and at pH 9 under hypoxic conditions to improve the solubility and stability of the 2‐Se‐nucleobases in aqueous media. To optimize the conversion, the reaction equilibria in analytical transglycosylation reactions were studied. The equilibrium constants of phosphorolysis of the 2‐Se‐pyrimidines were between 5 and 10, and therefore differ by an order of magnitude from the equilibrium constants of any other known case. Hence, the thermodynamic properties of the target nucleosides are inherently unfavorable, and this complicates their synthesis significantly. A tenfold excess of sugar donor was needed to achieve 40−48 % conversion to the target nucleoside. Scale‐up of the optimized conditions provided four Se‐containing nucleosides in 6–40 % isolated yield, which compares favorably to established chemical routes.

## Graphical abstract

**Se for yourself**: We present a biocatalytic synthesis of selenium‐containing pyrimidine nucleosides. Optimization of the reaction conditions improved the solubility and stability of the sensitive starting materials as well as previously observed low conversions. This approach offers a concise alternative to traditionally employed multistep routes to these compounds.

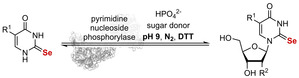

## Introduction

Selenium derivatization is a powerful tool for structure and function studies of nucleic acids. The distinct steric and electronic properties of selenium have facilitated the X‐ray structural analysis of DNA and RNA as well as their interactions with proteins.[^1^, ^2^, ^3^] Notably, this technique has been applied for the investigation of ribozymes,^[4]^ riboswitches,^[5]^ homo‐DNA,^[6]^ and for HIV‐1 drug discovery.^[7]^ Besides their application in X‐ray crystallography, selenium‐modified nucleic acids are potential therapeutics for the treatment of cancer,[^8^, ^9^] as well as viral[^10^, ^11^, ^12^] or bacterial infections.^[13]^ In addition, they have attracted interest as diagnostic agents.^[14]^ Several types of selenium derivatization of nucleic acids have been described which include replacement of an oxygen atom by selenium either in the nucleobase, the sugar moiety or in the phosphate groups of a nucleoside or nucleotide (see ref. [1]–[3] for reviews).

**Scheme 1:**
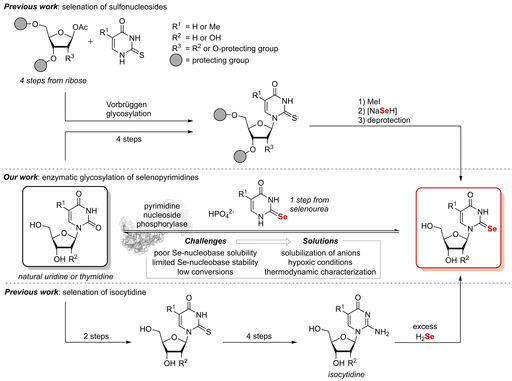
Approaches to the synthesis of 2‐Se pyrimidine nucleosides. Previous work has established the introduction of selenium by a methylated sulfonucleoside or selenation of isocytidine. Our work aims for biocatalytic direct glycosylation of a selenium‐containing nucleobase.

Despite their demand in chemical biology, a high‐yielding and sustainable synthesis of Se‐containing nucleosides has not yet been described. Previous work has established that 2‐Se pyrimidine nucleosides can be accessed either from methylated sulfo‐nucleosides or via selenation of isocytidine (Scheme 1).[^15^, ^16^, ^17^, ^18^, ^19^, ^20^, ^21^] However, these chemical syntheses have several drawbacks, including the need for multistep routes starting from a natural nucleoside, as well as an unfavorable atom economy and the extensive use of organic solvents.

Nucleoside phosphorylases (NP)‐catalyzed reactions offer a valuable alternative to chemical synthesis routes. NPs perform the reversible phosphorolysis of nucleosides to pentose‐1‐phosphates and nucleobases. By coupling the enzymatic phosphorolysis of a sugar donor with the glycosylation of a free nucleobase *in situ*, nucleosides can formally be synthesized directly from their corresponding nucleobases. The application of these enzymes resolves the need for protecting groups, laborious preparation as well as isolation of sugar synthons,^[22]^ and furthermore greatly reduces the use of toxic organic solvents by employing aqueous systems[^23^, ^24^] (for reviews, see refs. [25] and [26]).

In a proof‐of‐concept study, we recently described a protecting group‐free biocatalytic route for the synthesis of Se‐containing pyrimidine nucleosides (Scheme 1).^[27]^ We demonstrated on an analytical scale that 2‐ and 4‐Se‐substituted pyrimidine nucleosides can be prepared via transglycosylation reactions catalyzed by thermostable nucleoside phosphorylases (NPs).

This proof‐of‐concept study, however, revealed that several challenges remained to be addressed for the biocatalytic synthesis of Se‐containing nucleosides using NPs to transition from our previous report to a synthetically useful procedure. In recently published works, we explored the equilibrium thermodynamics of NP‐catalyzed reactions and applied principles of thermodynamic control to the improvement of conversions and yields in one‐ and two‐step systems.[^28^, ^29^] These groundworks were now applied for the rational yield optimization of the synthesis of 2‐Se‐nucleobases. Further challenges include the poor solubility of the free Se‐nucleobases in water, which has so far restricted substrate loading to approximately 1 mM. Furthermore, low substrate conversion was observed,^[27]^ indicating that the reaction equilibria of these reactions might be unfavorable. Additionally, prolonged reaction times have been shown to cause unwanted side reactions such as oxidation and deselenation of the starting material,[^27^, ^30^, ^31^, ^32^] resulting in heavy losses of the targeted product. Finally, our previous report included only non‐optimized reactions on an analytical scale and no product isolation, which limited product (and by‐product) characterization.

In this work, we addressed these obstacles by adjusting the reaction conditions to accommodate for the solubility and stability of Se‐nucleobases in the biocatalytic synthesis of 2‐Se‐pyrimidine nucleosides. Working under hypoxic conditions prevented deselenation and oxidative loss of yield, while an increased reaction pH allowed for higher substrate loading enabled by deprotonation of the Se‐nucleobases. A thermodynamic characterization of the reaction equilibria via analytical transglycosylations revealed equilibrium constants well outside the known range of similar nucleosides and guided our choice of suitable reaction conditions to maximize the product yields. This report presents improved yields compared to established procedures and describes the first biocatalytic synthesis of 2‐Se‐pyrimidine nucleosides.

## Results and Discussion

### Optimization of the reaction conditions to improve substrate loading of the 2‐Se‐bases

**Scheme 2:**
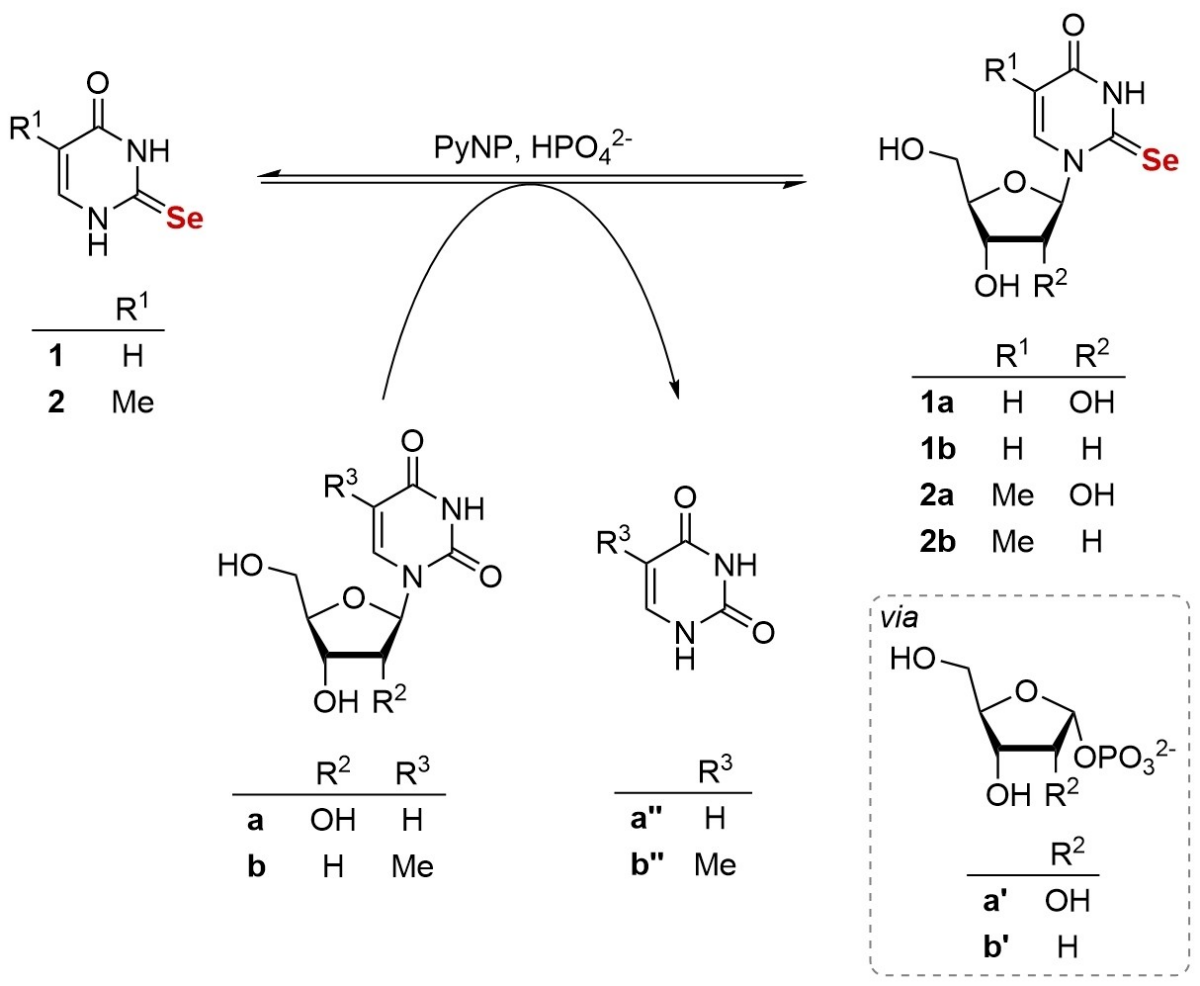
Biocatalytic synthesis of 2‐selenopyrimidine nucleosides in a transglycosylation reaction with a thermostable pyrimidine nucleoside phosphorylase (PyNP) as biocatalyst.

We aimed to access the selenium‐modified nucleosides **1 a**–**2 b** in a biocatalytic one‐pot approach using thermostable NPs (Scheme 2). For this study we selected the commercially available pyrimidine nucleoside phosphorylase Y04^[33]^ due to its thermostability and broad substrate spectrum. Analytical‐scale experiments in our previous report^[27]^ indicated that these compounds can, in principle, be synthesized via transglycosylation starting from the natural nucleosides uridine or thymidine and a selenium‐containing nucleobase.

For synthetic applications, a higher substrate loading than previously employed (1 mM at pH 7) would be highly desirable to reduce solvent use and facilitate purification. Based on the p*K*_a_ of 2‐Se‐uracil (**1**; 7.18 at RT)^[34]^ we hypothesized that a slightly higher reaction pH would facilitate dissolution of **1** and 2‐Se‐thymine (**2**) as their corresponding anions. As it was not available from the literature, we determined the p*K*_a_ of **2** through analysis of its UV absorption spectra at different pH values and found a similar value (7.49±0.01, Figure S1 in the Supporting Information) to that of **1**. Hence, compared to the natural nucleobases, the Se‐containing analogues were more acidic.[^16^, ^34^] Next, we investigated the solubility of **1** and **2** at pH values below and above their p*K*_a_ to see if a pH shift would allow higher substrate loading. At pH 7, **1** was not soluble at 10 mM, as indicated by the presence of precipitate (Figure S2). In contrast, full dissolution was observed at pH 9, confirming that a higher reaction pH can increase the solubility of the free nucleobase through deprotonation and solvation of the corresponding salt. Nonetheless, this effect was modest, as only **2** was fully soluble at 20 mM at pH 9 and RT.

A reaction temperature of 80 °C was initially chosen for the synthesis of Se‐modified nucleosides as higher reaction rates are observed and it can be assumed that a high reaction temperature would further improve the solubility of **1** and **2**. Initial studies, however, revealed product losses during the synthesis of 2’‐deoxyribonucleosides (data not shown) which can be ascribed to the hydrolysis of the transglycosylation intermediate **b′** at higher temperatures.[^23^, ^35^] Hence, reaction temperatures of 80 °C were applied for the synthesis of ribonucleosides, while 60 °C was chosen for reactions involving 2’‐deoxynucleosides.

As the pH and temperature might influence the activity of PyNP Y04, we determined specific activities of the enzyme at pH 7 or 9. To this end, we performed phosphorolysis reactions (Figure S3A) with the sugar donors uridine (**a**) and thymidine (**b**) in a MOPS (pH 7) or glycine buffer (pH 9). PyNP Y04 was active under all conditions and, for instance, catalyzed the phosphorolysis of **a** at 80 °C with activities of approximately 80 U mg^−1^ (Figure S3B). We additionally performed the glycosylation of **2** with the sugar phosphate **a′** to confirm that the higher reaction pH did not inhibit the second reaction step (reverse phosphorolysis). We observed no significant difference in glycosylation activity at pH 7 or 9, indicating that the deprotonated Se‐base is well‐accepted by the enzyme (Figure S4). Taken together, these data show that a reaction pH of 9 improves the solubility of **1** and **2** and thus permits higher substrate loadings, while still allowing excellent enzymatic activity, with reaction temperatures of 60 or 80 °C presumably facilitating additional solubility.

### Hypoxic conditions for the stability of the 2‐Se‐bases

**Figure 1:**
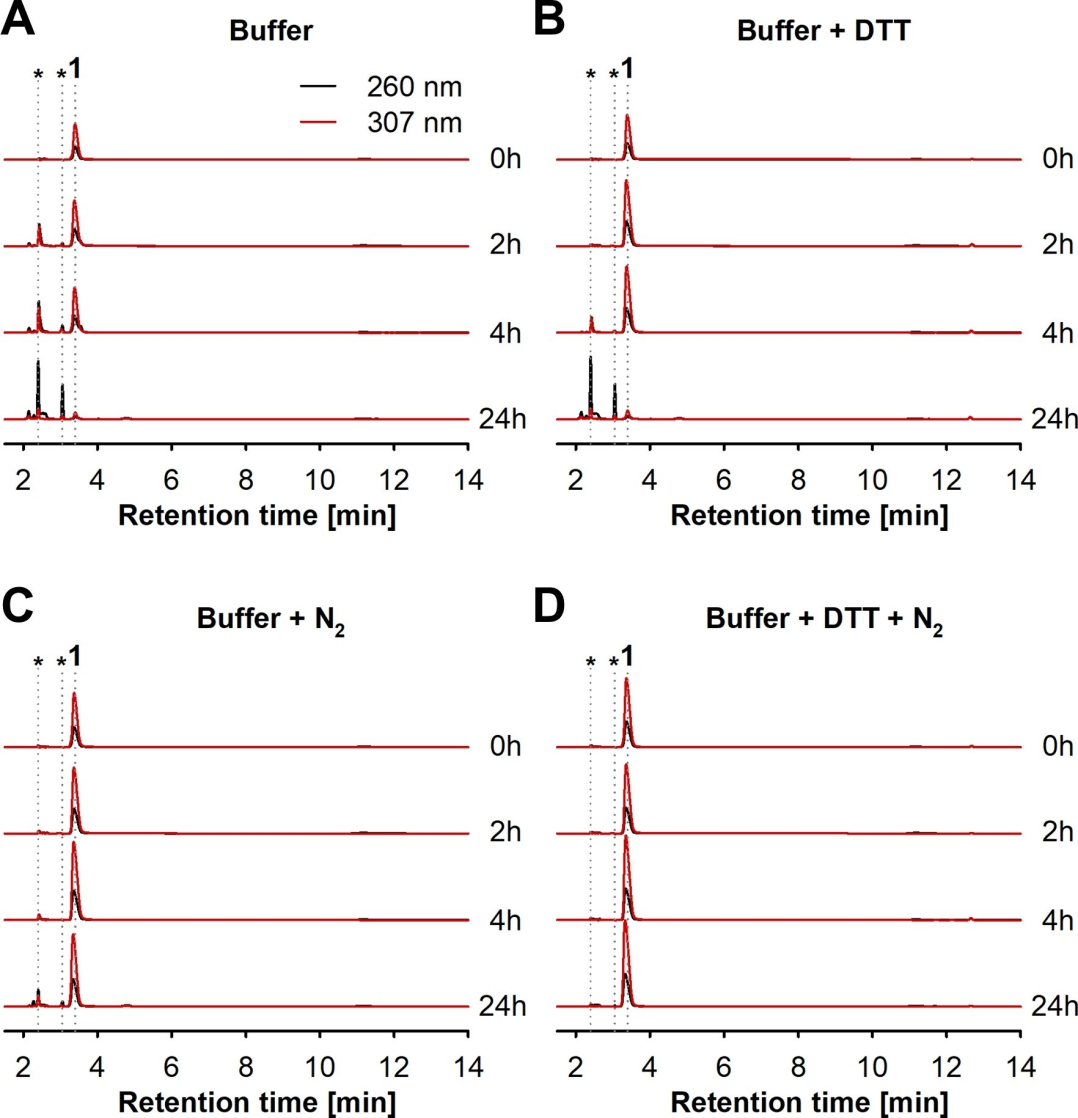
Stability of 5 mM **1** at 80 °C in 50 mM glycine/NaOH buffer pH 9 (A) without additives, (B) with 5 mM DTT, (C) saturated with nitrogen and (D) a combination of 5 mM DTT and nitrogen. Samples were analyzed at 260 (black) and 307 nm (red). The typical retention time of **1** is 3.4 min, and the degradation products appear at 2.4 and 3 min. Similar results were obtained for **2** (Figure S5). See ref. [36] for raw data.

The application of an alkaline reaction environment and high temperatures guided us to explore the stability of the 2‐Se‐bases under these rather harsh conditions. In early experiments we noticed significant oxidation of the starting materials **1** and **2** which manifested itself as a loss of substrate and product, as observed by HPLC, as well as discoloration of the reaction mixtures from colorless to red and black (data not shown). To prevent oxidation and deselenation, reducing agents,[^30^, ^31^, ^32^] such as dithiothreitol (DTT) and ascorbic acid, and inert gases^[17]^ like argon and nitrogen have previously been applied in chemical syntheses and redox studies. This led us to examine whether and for how long the stability of the 2‐Se‐bases can be increased by the application of DTT and/or a nitrogen atmosphere, which appeared compatible with our reaction system. To this end, we incubated **1** and **2** in different buffer systems (glycine/NaOH pH 9, with or without DTT and with or without a nitrogen atmosphere) at 80 °C and analyzed samples at different time points to check for the integrity of the starting material. Without any additives, oxidation products were already detected after 2 h, with most of the Se‐base being degraded after 24 h (Figures 1A and S4). The addition of DTT had a slightly conserving effect, as no oxidation was apparent after 2 h, but significant deselenation was apparent after 4 and 24 h (Figure 1B and S5). In contrast, saturation of the solution with nitrogen almost completely prevented oxidation of **1** and **2**, with only minimal oxidation product being detectable after 24 h (Figure 1C and S5). As expected, combining DTT and nitrogen prevented oxidation completely and conserved the starting material for 24 h (Figures 1D and S5).

Having established that DTT and nitrogen aid in stabilization of **1** and **2**, we questioned if these conditions would affect the activity of PyNP Y04. Therefore, we performed phosphorolysis experiments at 80 °C with either **a** in nitrogen‐saturated buffer or the model substrate 5‐iodouridine (**c**) in buffer with 5 mM DTT, as the latter nucleoside allowed us to obtain UV spectra that could be deconvoluted despite the heavy background absorption of DTT at lower wavelengths (see Figure S3 of ref. [37]). In contrast to the comparable activity observed in nitrogen‐saturated buffer, the application of DTT caused a≈40 % drop in enzymatic activity with our model substrate **c** (160–95 U mg^−1^; Figure S3B). However, due to the prevention of oxidation by DTT, we considered this decrease in enzymatic activity tolerable and proceeded with these conditions. In summary, the application of hypoxic conditions combined with the reducing agent DTT improved the stability of **1** and **2** in aqueous solution with no detectable degradation after 24 h at 80 °C, while only slightly affecting the enzymatic activity.

Transglycosylations were performed in a total volume of 1 mL in 50 mM glycine/NaOH pH 9 and 5 mM DTT at 60 °C (**b**) or 80 °C (**a**). [a] 1 mM 2‐Se‐nucleobase (**1** or **2**), 50.4 μg mL^−1^ (ca. 4 U) PyNP Y04, 5 mM sugar donor (**a** or **b**). [b] 1 mM 2‐Se‐nucleobase (**1** or **2**), 50.4 μg mL^−1^ (ca. 4 U) PyNP Y04, 10 mM sugar donor (**a** or **b**). [c] 5 mM 2‐Se‐nucleobase (**1** or **2**), 24.6 μg mL^−1^ (ca. 2 U) PyNP Y04, 50 mM sugar donor (**a** or **b**). Experimental conversion was determined by HPLC. Equilibrium constants were calculated with the experimental data of the fivefold sugar donor excess. Predictions of the conversion using tenfold sugar donor excess were carried out as described previously.^[28]^ See ref. [38] for raw data and calculations.

### Optimization of the enzymatic synthesis of 2‐Se‐pyrimidine nucleosides based on thermodynamic calculations

With hypoxic and alkaline reaction conditions in hand which served well to ensure solubility and stability of the nucleobases **1** and **2**, we turned our attention to the improvement of the previously observed low conversions.^[27]^ Recent work from our group had demonstrated the application of analytical‐scale experiments and thermodynamic calculations for the yield optimization in NP‐catalyzed transglycosylations.^[29]^ Therefore, we sought to transfer the same principles which had succeed for the synthesis of dihalogenated nucleosides[^28^, ^29^] to the preparation of Se‐pyrimidines to obtain improved yields. As nucleoside phosphorolysis (and consequently also the reverse reaction, glycosylation) is a thermodynamically controlled reaction, one may calculate the equilibrium state of transglycosylations via the corresponding equilibrium constants. Thus, when both equilibrium constants in the system are known, conversions can be optimized *in silico* to suggest conditions that enable the desired extent of product formation (for further details, see ref. [28]).

**Figure 2:**
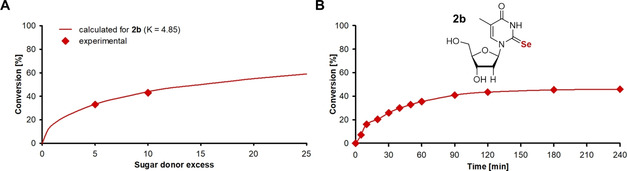
Optimization of the enzymatic synthesis of **2 b**. Transglycosylations were performed in a total volume of 1 mL by using (A) 1 or (B) 5 mM **2** and 5 mM DTT in 50 mM glycine/NaOH pH 9 at 60 °C with either (A) a five‐ and tenfold or (B) a tenfold excess of **b** compared to the nucleobase **2**. Final concentrations of PyNP Y04 of 50.4 μg mL^−1^ (ca. 4 U, A) and 24.6 μg mL^−1^ (ca. 2 U, B) were applied. Experimental conversion was determined by HPLC as the conversion of **2** to **2 b**. The equilibrium constant of phosphorolysis was calculated based on the experimental data of the fivefold sugar donor excess (A). Predictions of the conversion using different sugar donor to nucleobase ratios were carried out as described previously.^[28]^ Similar results were obtained for the other products (Table 1). See ref. [38] for raw data and calculations.

Equilibrium constants for the phosphorolysis of the sugar donors have been described recently for a broad temperature range,^[39]^ but the corresponding values for 2‐Se‐nucleosides have not been reported yet. As these constants are required for yield optimization through equilibrium thermodynamics, we performed small‐scale transglycosylation reactions to determine these indirectly. Using a fivefold excess of the sugar donor over the Se‐nucleobase and 0.09 equivalents of phosphate, equilibrium conversions between 30 and 35 % were observed (Table 1, Figure 2A for **2 b** as a visual example). This yielded equilibrium constants of the phosphorolysis of the Se‐nucleosides **1 a**–**2 b** in the range of 5 to 10 (Table 1). Interestingly, these equilibrium constants surpass those of other natural and modified pyrimidine nucleosides (0.1–0.8)[^28^, ^39^, ^40^] by around an order of magnitude. While the reason for these high equilibrium constants is unclear to date, we hypothesize that the increased electron density near the glycosylation site may contribute to a weaker C1’−N1 bond.

**Table 1:** Equilibrium state thermodynamic calculations were used to determine appropriate reaction conditions for the synthesis of **1 a**–**2 b**.

Product	Product formation [%] at equilibrium for 5‐fold sugar donor excess^[a]^	Equilibrium constant of phosphorolysis	Product formation [%] at equilibrium for 10‐fold sugar donor excess		
			calculated^[a]^	experimental (1 mM nucleobase)^[b]^	experimental (5 mM nucleobase)^[c]^
**1a**	30.5	9.37	39	40	46 (4 h)
**1b**	30	6.07	40	40	39 (3 h)
**2a**	35	6.75	44	47	48 (4 h)
**2b**	33	4.85	44	43	45 (3 h)

Transglycosylations were performed in a total volume of 1 mL in 50 mM glycine/NaOH pH 9 and 5 mM DTT at 60 °C (**b**) or 80 °C (**a**). [a] 1 mM 2‐Se‐nucleobase (**1** or **2**), 50.4 μg mL^−1^ (ca. 4 U) PyNP Y04, 5 mM sugar donor (**a** or **b**). [b] 1 mM 2‐Se‐nucleobase (**1** or **2**), 50.4 μg mL^−1^ (ca. 4 U) PyNP Y04, 10 mM sugar donor (**a** or **b**). [c] 5 mM 2‐Se‐nucleobase (**1** or **2**), 24.6 μg mL^−1^ (ca. 2 U) PyNP Y04, 50 mM sugar donor (**a** or **b**). Experimental conversion was determined by HPLC. Equilibrium constants were calculated with the experimental data of the fivefold sugar donor excess. Predictions of the conversion using tenfold sugar donor excess were carried out as described previously.^[28]^ See ref. [38] for raw data and calculations.

Next, we used thermodynamic calculations to predict the maximal conversions in transglycosylation reactions employing different sugar donor excesses (Figure 2A, Table 1). These predictions revealed that the high equilibrium constants of phosphorolysis of the target nucleosides limited the maximum conversion severely. For example, to obtain 50 % conversion to the Se‐nucleoside **1 a**, an approximately 20‐fold excess of the sugar donor **a** would be necessary. However, such a high sugar donor excess is not suitable for preparative experiments due to high substrate costs and waste. In addition, we expected that this would also prevent an efficient workup and purification. Thus, we applied a sugar donor excess of 10 as a compromise to the aforementioned issues and maximal conversion. Although other sugar donors such as 5‐ethynyluridine or 7‐methylguanosine or direct glycosylation approaches with the sugar phosphates **a’** or **b’** might provide higher conversions than the application of natural uridine (**a**) and thymidine (**b**), these starting materials are considerably more expensive and would render this synthetic approach unfeasible (Table S1). Therefore, we decided to employ cheaply available **a** and **b** as sugar donors, which conveniently also offer some of the more favorable equilibrium constants of phosphorolysis among the natural nucleosides.^[39]^

Finally, we confirmed our predictions for 10 equivalents of **a** or **b** and evaluated the reaction times until equilibrium in analytical‐scale experiments under optimized conditions. The predicted extent of product formation was observed for all four 2‐Se‐nucleosides (Figure 2B, Table 1) with reaction completion occurring within 3 to 4 h. Taken together, our thermodynamic characterization of the reaction system revealed unfavorable equilibrium constants of phosphorolysis of all Se‐containing target nucleosides, severely limiting the maximum conversion achievable in transglycosylation reactions. However, a tenfold excess of the sugar donors allowed for 40−48 % conversion for all Se‐nucleosides.

### Enzymatic synthesis and purification in semipreparative scale

After optimizing the synthesis under hypoxic and alkaline reaction conditions in small‐scale experiments, we aimed to synthesize and purify the four 2‐selenopyrimidines in semi‐preparative scale. Therefore, the optimized reaction conditions were up‐scaled to a volume of 50 mL with 5 mM 2‐Se‐nucleobase. The observed product formations in the larger scale of 41–47 % after 3 (**2 b**), 4 (**1 b**) or 5 h (**1 a, 2 a**; Table 2) were in good accordance with the small‐scale experiments. Purification of the target compounds from these reaction mixtures, however, proved rather challenging due to the presence of large quantities of unreacted sugar donor. In fact, our attempts to purify any of the products by preparative HPLC were unsuccessful as the obtained material persistently contained sugar donor starting material. Therefore, we applied an initial silica chromatography step on normal phase to remove most of the sugar donor, followed by a second purification step via preparative HPLC. Using this two‐step process, we obtained 4.9–29.5 mg of the target nucleosides, corresponding to isolated yields of 6–40 % (Table 2, Figures S6–S9). Despite the incomplete conversions and product losses during purification, our biocatalytic route compares favorably to its chemical counterparts where yields of less than 10 % are typically achieved.^[27]^ Clearly, purification presents a major bottleneck of the route employed herein, yet this is largely owed to the necessity of employing large sugar donor excesses to drive the reaction. We expect that future efforts to alternative transglycosylation protocols will facilitate the purification process significantly by providing easier‐to‐separate crude products. Work to this end is ongoing in our laboratory.

**Table 2:** Yields and purity of 2‐Se‐pyrimidine nucleosides.

Product	Conversion	Isolated yield	Purity
	[%] (*t* [h])	[mg] ([%])	[%]^[a]^
**1 a**	44 (5)	4.9 (6.3)	98
**1 b**	41 (4)	29.5 (40.5)	94
**2 a**	47 (5)	20.8 (25.9)	99
**2 b**	45 (3)	9.6 (12.5)	99

The 50 mL reaction mixture consisted of 5 mM 2‐Se‐base (**1** or **2**), 50 mM sugar donor (**a** or **b**), 5 mM DTT and 24.6 μg mL^−1^ (ca. 98 U) PyNP Y04 in 50 mM glycine/NaOH pH 9 saturated with nitrogen. The reaction temperature was 60 °C (**1 b**, **2 b**) or 80 °C (**1 a**, **2 a**). [a] Purity was determined via analytical HPLC. See the Supporting Information for raw data.

## Conclusion

This work presents an optimized biocatalytic synthesis routes for 2‐Se‐pyrimidine nucleosides via transglycosylation from uridine or thymidine. Initial experiments and thermodynamic characterization of the reaction system revealed that the thermodynamic properties of the target nucleosides are very unfavorable and that the applied nucleobases are inherently poorly soluble in almost all solvents. Therefore, >40 % isolated yield and substrate loadings of 5 mM represent major improvements compared to previous synthetic efforts and likely also represent a ceiling from a thermodynamic standpoint. After adjusting the reaction conditions the target nucleosides on a small scale, four selenium‐containing pyrimidine nucleosides were obtained in scale‐up experiments where alkaline and hypoxic conditions, as well as high temperatures, enabled sufficient solubility and stability of the starting materials. Although the present route delivers improved yields compared to previous efforts, product purification currently represents a major bottleneck and needs to be addressed in future studies.

## Experimental Section

**General information**: All chemicals and solvents were of analytical grade or higher and purchased, if not stated otherwise, from Sigma‐Aldrich (Steinheim, Germany), Carl Roth (Karlsruhe, Germany), TCI Deutschland (Eschborn, Germany), Carbosynth (Berkshire, UK) or VWR (Darmstadt, Germany). 2‐Se‐uracil (**1**) and 2‐Se‐thymine (**2**) were prepared according to literature procedures.^[34]^

Thermostable nucleoside phosphorylase Y04^[33]^ was obtained from BioNukleo GmbH (Berlin, Germany) and used as recommended by the manufacturer. The thermostable PyNP Y04 originates from a hyper‐thermophilic bacterium and was heterologously expressed in *Escherichia coli* and purified by affinity chromatography. The provided stock solution (1.12 mg mL^−1^) was stored at 4 °C. The enzyme originates from a thermophilic bacterium with an optimum growth temperature of 80 °C.

All UV/Vis absorption spectra were recorded with a BioTek PowerWave HT plate reader using UV/Vis‐transparent 96‐well plates (UV‐STAR F‐Bottom #655801, Greiner Bio‐One). All raw and calculated data described in this article are freely available from an external online repository.^[38]^

**Determination of the acid dissociation constant (p*K***_**a**_ **) of 2‐Se‐modified nucleobases**: The p*K*_a_ of **1** and **2** was determined by analysis of their UV/Vis absorption spectra between pH 4 and pH 10 at RT. In a total volume of 10 mL, the nucleobases were dissolved to a concentration of 100 μM in 50 mM MOPS buffer (initially pH 9). Desired pH values were adjusted with HCl and NaOH and samples of 200 μL were transferred to a UV/Vis‐transparent 96‐well plate to record the UV absorption spectra in the range of 250 to 350 nm in steps of 1 nm. The spectra were analyzed via spectral unmixing as previously described[^37^, ^41^] using a fully protonated (pH 4) and a fully deprotonated (pH 10) spectrum as substrate and product reference for the deprotonation reaction, as spectra obtained near these pH values displayed identical shape (indicating no further reaction). For the determination of the p*K*_a_, the experimental data were fitted to Equation (1), whereby the pH was set as the input variable, α as the dependent variable and p*K*_a_ as the parameter to fit.^[42]^

(1) $$\alpha =\frac{x_{deprot}}{x_{deprot}+x_{prot}}=\;\frac{10^{pH-{pK}_{a}}}{1+10^{pH-{pK}_{a}}}$$

**Solubility of the 2‐Se‐modified nucleobases**: The solubility of **1** and **2** was tested in 50 mM MOPS/NaOH buffer pH 7 and 50 mM glycine/NaOH buffer pH 9 at RT. In a total volume of 0.3 to 10 mL, the nucleobases were dissolved to concentrations of 1, 5, 10 and 20 mM. The solutions were observed for the occurrence of precipitate.

**Stability of the 2‐Se‐modified nucleobases**: The stability of **1** and **2** was analyzed at 80 °C in Pyrex® glass tubes with screw cap. Therefore, the nucleobases were dissolved to a concentration of 5 mM in the following four buffers: a) 50 mM glycine/NaOH buffer pH 9, b) 50 mM glycine/NaOH buffer pH 9 with 5 mM 1,4‐dithioreitol (DTT), c) 50 mM glycine/NaOH buffer pH 9, saturated with nitrogen and d) 50 mM glycine/NaOH buffer pH 9 with 5 mM DTT, saturated with nitrogen. Samples were taken after 0, 2, 4 and 24 h, and diluted to a final concentration of 1 mM nucleobase with MeOH. After centrifugation (4 °C and 21 500 *g* for 20 min), samples were analyzed by analytical HPLC as described below.

**Enzyme activity assays**: The activity of PyNP Y04 was determined by performing phosphorolysis reactions with the substrates uridine (**a**), thymidine (**b**) and the model substrate 5‐iodouridine (**c**). Samples were analyzed by the UV/Vis spectroscopy‐based assay described recently.[^37^, ^41^] Briefly, a 500 μL reaction mixture consisting of 1 mM nucleoside and 50 mM K_2_HPO_4_ in 50 mM buffer (glycine/NaOH pH 9 or MOPS/NaOH pH 7) was preheated to the desired temperature (60 or 80 °C). The reaction was started by the addition of the enzyme (typically 10 μL of enzyme stock solution prediluted in 2 mM potassium phosphate buffer pH 7). Final concentrations of PyNP Y04 from 150 to 750 ng mL^−1^ were applied. At timely intervals, samples of 60 μL were taken and quenched in 450 μL 100 mM NaOH and 200 μL of the diluted sample was transferred to a UV/Vis‐transparent 96‐well plate to record the UV absorption spectra from 250 to 350 nm in steps of 1 nm. Spectral analysis was carried out as described previously[^37^, ^41^] with software^[43]^ and reference spectra^[44]^ freely available online.

To assay PyNP Y04 for the glycosylation of the Se‐nucleobase **2**, reactions consisting of 1 mM **2**, 10 mM **b’** and 70 μg mL^−1^ enzyme in 50 mM glycine/NaOH buffer pH 9 were monitored. For analysis, samples of 60 μL were withdrawn, quenched in an equal volume of MeOH and diluted with 400 μL 100 mM glycine/NaOH buffer pH 10. The experimental spectra were fitted with corresponding reference spectra of **2** and **2 b** obtained under the same conditions.

One unit (U) of enzyme activity was defined as the amount of the enzyme catalyzing the conversion of 1 μmol of substrate per minute under the described assay conditions.

**Optimization of the enzymatic synthesis of 2‐Se‐modified nucleosides**: The 2‐Se‐modified nucleosides **1 a**–**2 b** were accessed in a one‐pot transglycosylation reaction using the pyrimidine nucleoside phosphorylase PyNP Y04 as biocatalyst. The thermodynamic characterization was performed as described previously.[^28^, ^29^] Reaction equilibria were determined from reactions with 1 mM 2‐Se‐nucleobase (**1** or **2**), 5 mM sugar donor (**a** or **b**), 5 mM DTT, 50 mM glycine/NaOH buffer pH 9 and 50.4 μg mL^−1^ (ca. 4 U) PyNP Y04 in a total volume of 1 mL. The nitrogen‐saturated reaction mixtures were incubated at 60 °C for the deoxyribosides **1 b** and **2 b** and 80 °C for the ribosides **1 a** and **2 a** until reaction completion (indicated by no further product formation; within 30 min under these conditions). Samples were diluted to 1 mM sugar donor concentration in MeOH, centrifuged (4 °C, 21 500 g, 20 min) and analyzed by HPLC.

The equilibrium constants of phosphorolysis of the 2‐Se‐pyrimidine nucleosides were calculated via equilibrium state thermodynamics via numerical solutions. Nucleoside phosphorolysis is a tightly thermodynamically controlled reaction and transglycosylations behave as coupled equilibrium reactions as they comprise a forward and a reverse phosphorolysis. Thus, knowledge of the equilibrium constant of the sugar donor, the concentrations of the starting materials as well as the degree of conversion to the product nucleoside allows calculation of the equilibrium constants of phosphorolysis of the product nucleoside via numerical solutions of the system of coupled equilibrium constraints (see refs. [28] and [29]). These numerical solutions can either be obtained via the Python code described in our previous work^[45]^ or, more conveniently, the Excel sheet presented in the externally hosted Supplementary Information of this publication. Herein, we used the previously published equilibrium constants of phosphorolysis of the sugar donors **a** and **b** for calculation.[^39^, ^46^] Based on the obtained equilibrium constants, expected conversions for the 2‐Se‐nucleosides using tenfold sugar donor excess were calculated (see the externally hosted Supporting Information for details).[^38^, ^45^] To verify the theoretical calculations and to monitor the reaction progression, reactions with 5 mM 2‐Se‐base, 50 mM sugar donor, 5 mM DTT in 50 mM glycine/NaOH buffer pH 9 saturated with nitrogen and 24.64 μg mL^−1^ (ca. 2 U) PyNP Y04 in a total volume of 1 mL were performed at 60 °C for the deoxyribosides and 80 °C for the ribosides. Samples were diluted 50‐fold in MeOH, centrifuged (4 °C, 21,500 g, 20 min) and analyzed by HPLC.

**Analytical HPLC**: Analytical HPLC analyses were carried out with an Agilent 1200 series system equipped with an Agilent DAD detector using a Phenomenex (Aschaffenburg, Germany) reversed phase Kinetex EVO C_18_ column (250×4.6 mm). Samples were analyzed at two wavelengths (*λ*=260 nm, 307 nm) at 25 °C and a flow rate of 1 mL min^−1^. Isocratic elution was performed using 97 % 20 mM ammonium acetate buffer and 3 % acetonitrile for 7 min followed by a linear gradient to 60 % 20 mM ammonium acetate buffer and 40 % acetonitrile over 8 min. Afterwards, the initial conditions were restored and maintained for 4 min.

Conversions were determined by quantifying the 2‐Se‐nucleosides **1 a**–**2 b** and 2‐Se‐nucleobases **1** and **2** at 307 nm by using Equation (2), whereby $P_{X}$ is the peak area of compound X and *P*_total_ is the sum of all peak areas at 307 nm. Substrates and products were identified based on their retention time and UV absorption spectra by comparison to authentic standards. Typical retention times under these conditions were as follows: **1**: 3.4 min, **2**: 6 min, **a**: 3.6 min, **b**: 8.6 min, **a’’**: 3 min, **b’’**: 4.5 min, **1 a**: 7 min, **1 b**: 12 min, **2 a**: 12 min and **2 b**: 13 min. **a’** and **b’** are not UV active and cannot be detected with a DAD system.

(2) $$Conversion\;\left(X\right)\;\left[\%\right]=100\;\times \frac{P_{X}\;}{P_{total}\;}$$

**Synthesis of 2‐Se pyrimidine nucleosides on a semi‐preparative scale**: 2‐Se nucleosides were synthesized in a reaction volume of 50 mL consisting of 5 mM 2‐Se base (**1** or **2**), 50 mM sugar donor (**a** or **b**), 5 mM DTT, 50 mM glycine/NaOH buffer pH 9 and 24.6 μg mL^−1^ (**a**: 98.5 U, **b**: 97.4 U) PyNP Y04. The reaction mixture was saturated with nitrogen. Deoxyribosyl derivatives were prepared at 60 °C and ribosyl derivatives at 80 °C. The reactions were stopped after 3 to 5 h by a pH shift to 13 through addition of 10 M NaOH (monitored with a pH electrode). The pH was shifted back to 9 using 25 % HCl and proteins were removed by filtration at room temperature using a vacuum pump and 0.45 μm cellulose nitrate filters (Sartorius, Goettingen, Germany). The filtrate was saturated with nitrogen and stored at 4 °C until purification.

**Purification of 2‐Se pyrimidine nucleosides by Silica column chromatography**: The first purification step was performed at room temperature via column chromatography (10 % MeOH in CH_2_Cl_2_) using a Sigma‐Aldrich silica gel (pore size 60; mesh particle size 220–440; particle size 35–75 μm). The reaction mixture was adsorbed onto silica gel by addition of 1.8 g of silica to the aqueous reaction mixture and evaporation of the solution *in vacuo*. The resulting powder was stored at −20 °C until loading on a silica column for purification. Collected fractions were analyzed by TLC (10 % MeOH in CH_2_Cl_2_) using UV detection. Fractions containing the 2‐Se‐nucleoside were combined and dried under reduced pressure and stored at −20 °C until further purification by semi‐preparative HPLC.

**Purification of 2‐Se pyrimidine nucleosides by semi‐preparative HPLC**: 2‐Se‐nucleosides were further purified at room temperature using a Knauer HPLC system equipped with a Smartline Detector 2600 and an Azura P2.1 L pump. A reversed phase Kinetex® 5 μm Evo C_18_ column (250×21.2 mm) and a flow rate of 21.24 mL min^−1^ were used. Samples were analyzed at 210 nm. Deionized water and acetonitrile were applied as eluents, while the gradient was modified from the analytical method as summarized in Table S2. The product purified by silica chromatography was dissolved in deionized water (Table S2) and filtered either with a 0.45 μm PES syringe filter or with a 0.45 μm cellulose nitrate filter using a vacuum pump. The collected product fractions were kept on ice to prevent product degradation during the purification process. Collected 2‐Se‐nucleoside fractions were dried using a Christ Gamma 1–20 freeze‐dryer (Osterode am Harz, Germany).

**LC‐MS analysis**: For LC‐MS analysis, 0.1 mg sample was dissolved in 1 mL water in a HPLC vial. Samples were analyzed using a HPLC Agilent 1200 series system coupled to an ESI‐Orbitrap‐MS (Thermo Fisher LTQ Orbitrap XL). The LC analyses were carried out using a Grace reversed phase GROM‐Sil‐ODS‐4‐HE column (50×2 mm, 3 μm). Samples were analyzed at 20 °C and at three wavelengths (*λ*=215, 280, 350 nm). A flow rate of 0.3 mL min^−1^ was applied. The gradient was linearly increased from 80 % 0.1 % HCOOH in water and 20 % 0.1 % HCOOH in ACN to 100 % 0.1 % HCOOH in ACN in 10 min. The mobile phase composition was held for 3 min at 100 % 0.1 % HCOOH in ACN. Finally, the initial conditions were restored and maintained for 5 min. The raw data was analyzed using FreeStyle (Thermo Scientific).

### Compound characterization (also see Figures S6–S9)

*2‐Selenouridine (**1 a**)*: off‐white powder, *R*_f_=0.29 (CH_2_Cl_2_/MeOH 9 : 1); UV/Vis (4.5 % ACN in 20 mM NH_4_Ac pH 6.8): *λ*_max_ =307 nm; HRMS (ESI): *m/z* calcd for C_9_H_12_N_2_O_5_Se+H^+^: 308.9984 [*M*+H]^+^; found: 308.9986

*2’‐Deoxy‐2‐Selenouridine (**1 b**)*: yellow powder, *R*_f_=0.39 (CH_2_Cl_2_/MeOH 9 : 1); UV/Vis (26 % ACN in 20 mM NH_4_Ac pH 6.8): *λ*_max_ =307 nm; HRMS (ESI): *m/z* calcd for C_9_H_12_N_2_O_4_Se+H^+^: 293.0035 [*M*+H]^+^; found: 293.0036

*5‐Methyl‐2‐selenouridine (**2 a**)*: slightly cream‐colored powder, *R*_f_=0.38 (CH_2_Cl_2_/MeOH 9 : 1); UV/Vis (26 % ACN in 20 mM NH_4_Ac pH 6.8): *λ*_max_ =307 nm; HRMS (ESI): *m/z* calcd for C_10_H_14_N_2_O_5_Se+H^+^: 323.0141 [*M*+H]^+^; found: 323.0144

*2‐Selenothymidine (**2 b**)*: off‐white powder, *R*_f_=0.43 (CH_2_Cl_2_/MeOH 9 : 1); UV/Vis (30 % ACN in 20 mM NH_4_Ac pH 6.8): *λ*_max_ =307 nm; HRMS (ESI): *m/z* calcd for C_10_H_14_N_2_O_4_Se+H^+^: 307.0192 [*M*+H]^+^; found: 307.0194

## Supporting information

As a service to our authors and readers, this journal provides supporting information supplied by the authors. Such materials are peer reviewed and may be re‐organized for online delivery, but are not copy‐edited or typeset. Technical support issues arising from supporting information (other than missing files) should be addressed to the authors.

Supplementary
