# Supplementary material for: Optimized Biocatalytic Synthesis of 2‐Selenopyrimidine Nucleosides by Transglycosylation
Source: Chembiochem. 2021 Mar 31;22(11):2002–9. doi: 10.1002/cbic.202100067 (PMC8251958; doi:10.1002/cbic.202100067)
Supplement: Supplementary file 1 — Supplementary [file CBIC-22-2002-s001.pdf]

ChemBioChem

Supporting Information

Optimized Biocatalytic Synthesis of 2-Selenopyrimidine Nucleosides by Transglycosylation**

Katja F. Hellendahl+, Felix Kaspar+, Xinrui Zhou, Zhaoyi Yang, Zhen Huang, Peter Neubauer, and Anke Kurreck*

Author contributions	1
Data availability	1
Plots for pK_a determination	2
Solubility of the 2-Se-bases	2
Specific activity of PyNP Y04 in phosphorolysis reactions	3
Specific activity of PyNP Y04 for the synthesis of **2b** via direct glycosylation	4
Stability of **2**	4
Overview of the equilibrium constants and prices of potential sugar donors	5
Purification of 2-Se-nucleosides by semi-preparative HPLC	5
HPLC and MS data of **1a**	6
HPLC and MS data of **1b**	7
HPLC and MS data of **2a**	8
HPLC and MS data of **2b**	9
References	10

Author Contributions (with definitions as recommended by Brand et al.[1])

Conceptualization, K.F.H., F.K., P.N. and A.K.; methodology, K.F.H., F.K. and A.K.; software, F.K.; validation, K.F.H.; formal analysis, K.F.H and F.K.; investigation, K.F.H and F.K.; resources, X.Z., Z.Y., Z.H., A.K and P.N.; writing – original draft, K.F.H and F.K; writing – review and editing, K.F.H., F.K., X.Z., Z.Y., Z.H., A.K. and P.N.; visualization, K.F.H, F.K. and A.K., supervision, A.K. and P.N.; project administration K.F.H, F.K., A.K. and P.N.; Funding acquisition, A.K. and P.N.

All authors have read and agree to the published version of the manuscript.

Data availability

All data depicted visually in the items in the main text as well as in the Supplementary Information is available from an externally hosted Supporting Information.[2]

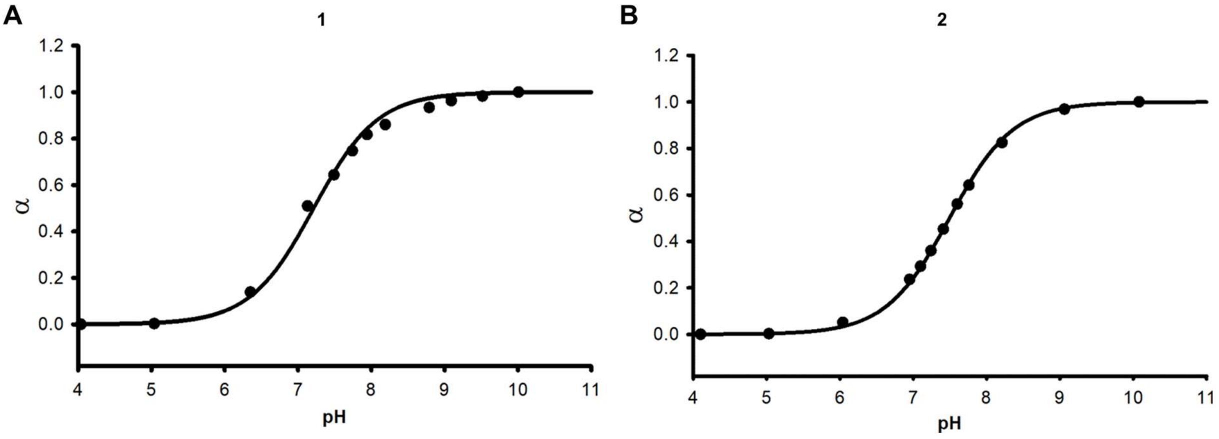

Figure S1. Plots for the pK_a determination of **1** (A) and **2** (B). The 2-Se-nucleobases were dissolved to a concentration of 100 µM in 50 mM MOPS buffer. The pH was adjusted with HCl and NaOH at RT. Samples were analysed by spectral unmixing[2] using the isosbestic points of 282 nm for **1** and 279 nm for **2**. The pK_a for **1** was 7.21 ± 0.03 and 7.49 ± 0.006 for **2**.

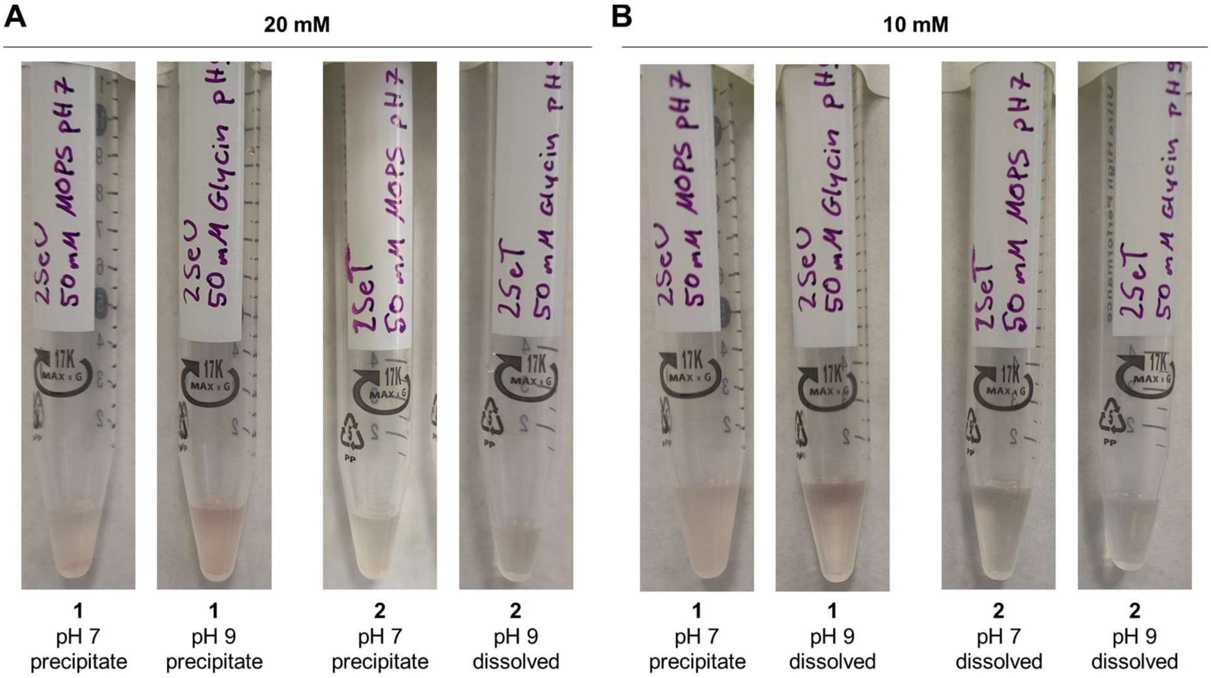

Figure S2. Solubility of 20 mM (A) and 10 mM (B) of **1** and **2** in 50 mM MOPS pH 7 and 50 mM Glycine pH 9 at RT.

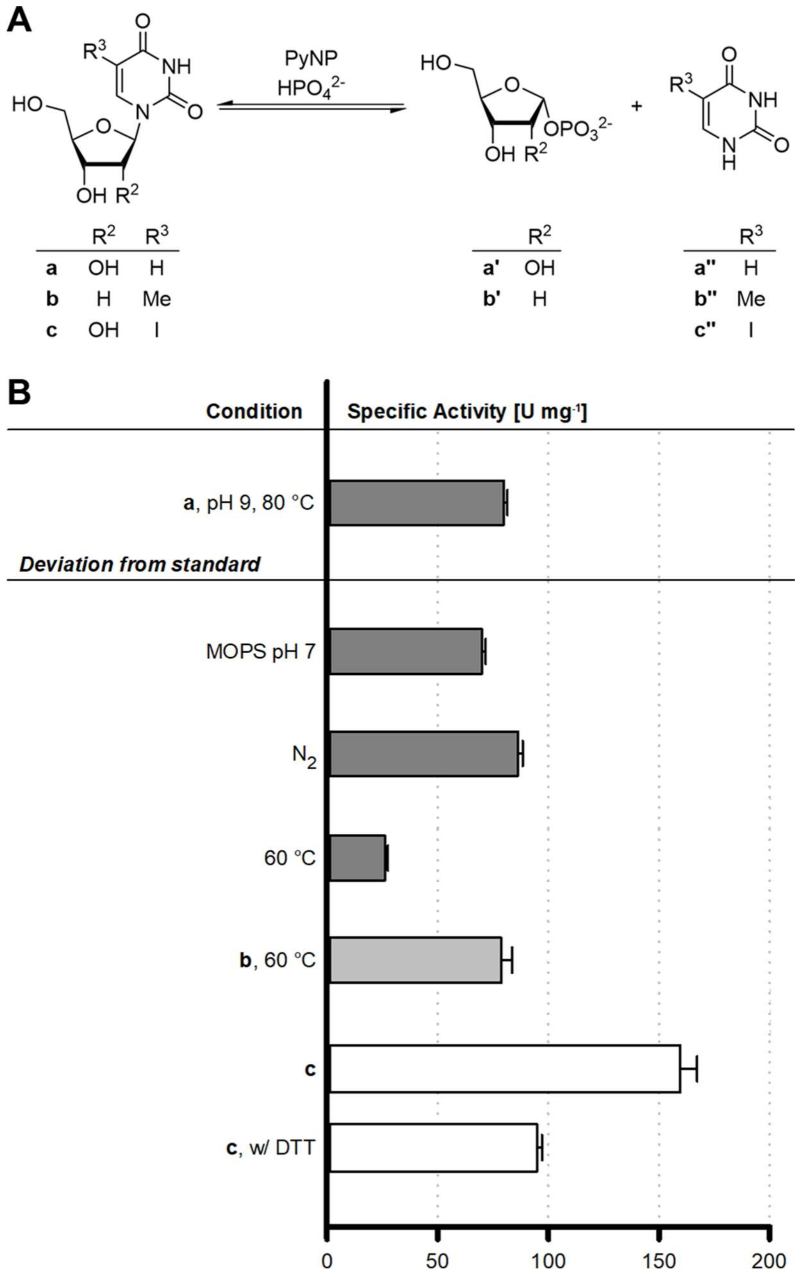

Figure S3. Specific activity of PyNP Y04 in phosphorolysis reactions (A) under different conditions (B). Standard phosphorolysis reactions were performed with 1 mM **a**, 50 mM K_2HPO_4 in 50 mM glycine/NaOH buffer pH 9 in a total volume of 0.5 mL at 80 °C. Final concentrations of PyNP Y04 of 150 to 750 ng mL^{-1} were applied. To study the impact of substrates (**b**, **c**), temperature and reducing conditions on the enzyme activity. Reactions were performed at 60°C and pH 7, under N_2 atmosphere or with the addition of 5 mM DTT. Samples were analysed via spectral unmixing.[2]

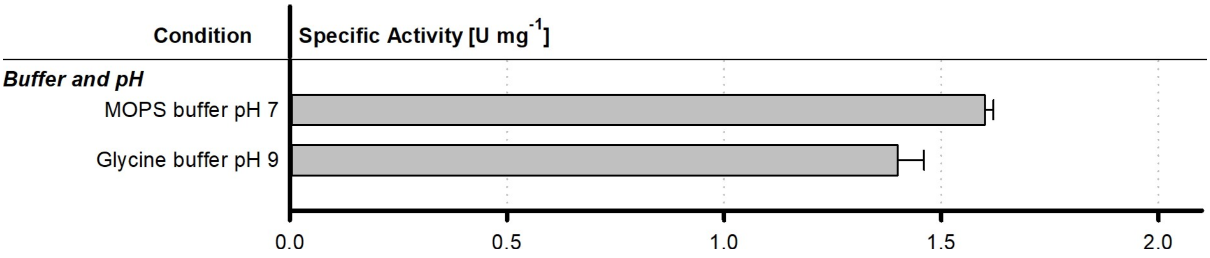

Figure S4. Specific activity of PyNP Y04 for the synthesis of **2b** via direct glycosylation. Reactions were performed with 1 mM **2**, 10 mM **b'** and 70 µg mL^{-1} PyNP Y04 in 50 mM glycine/NaOH pH 9 in a total volume of 250 µL at 60 °C. Samples were analysed via spectral unmixing.[2]

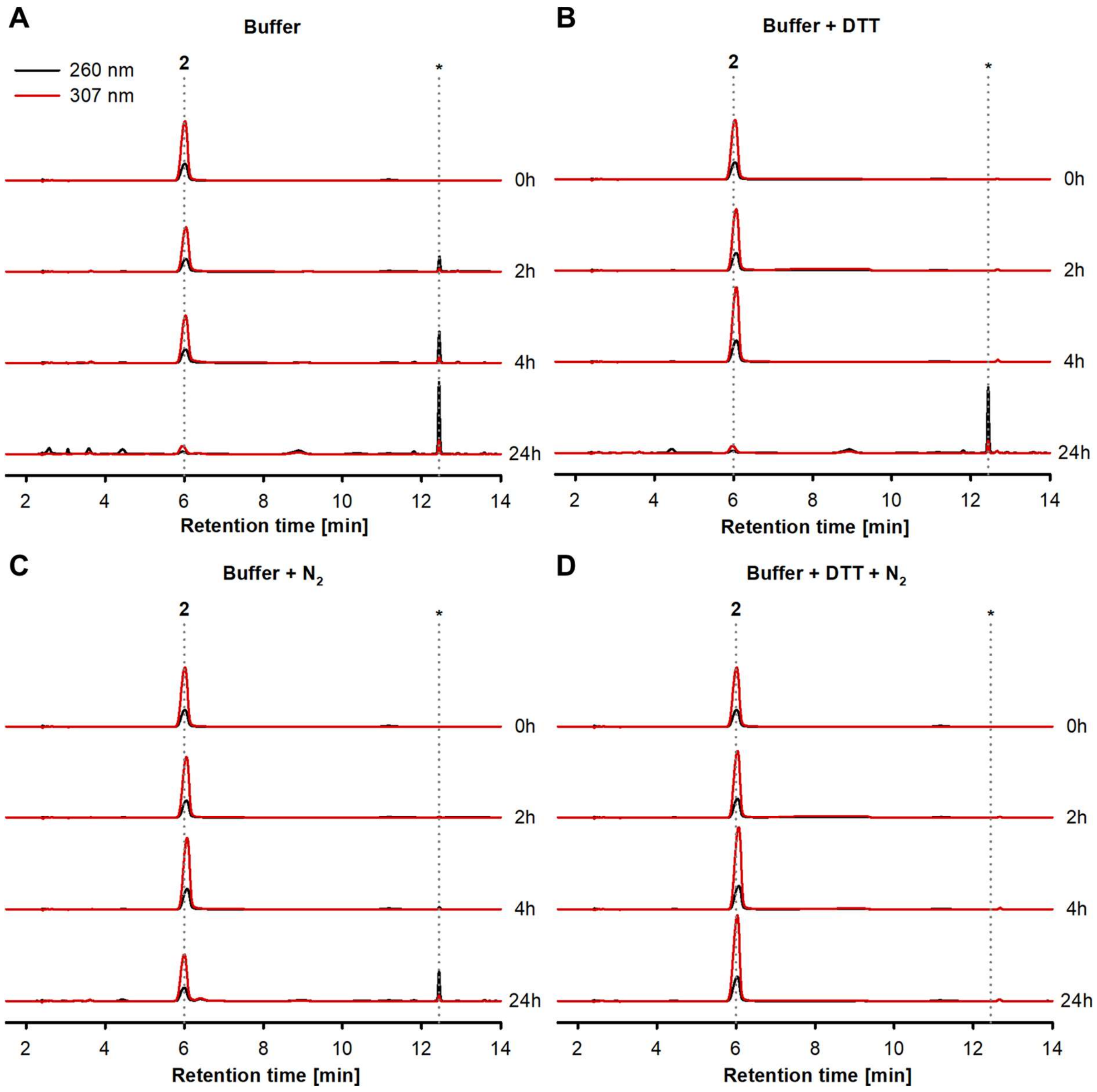

Figure S5. Stability of 5 mM **2** at 80 °C in 50 mM glycine/NaOH buffer pH 9 without additives (A), with 5 mM DTT (B), saturated with nitrogen (C) and the combination of 5 mM DTT and nitrogen (D). Samples were diluted to 1 mM in MeOH and analysed at 260 nm (black) and 307 nm (red) by HPLC. Retention time of 2 is 6 min and the degradation peak is at 12.44 min.

Table S1 Overview of equilibrium constants and prices of potential sugar donors.

	Sugar donor	Equilibrium constant	Price [€ g^{-1}][b]
**Ribose**	Uridine	0.18[a]	4.5 (100 g 450 €)
	5-Ethynyluridine	0.61[a]	11,000 (5 mg 55 €)
	7-Methylguanosine		1188 (250 mg 297 €)
**Deoxyribose**	Thymidine	0.15[a]	13.52 (25 g 338 €)
	5-Ethynyl-2'-deoxyuridine	0.35[a]	1948 (500 mg 974 €)

[a] at 40 °C from [3–5]

[b] Prices were calculated from the biggest pack size available for Germany on the Sigma-Aldrich website. Last access 28.09.2020.

Table S2 Purification of 2-Se-nucleosides by semi-preparative HPLC.

Product	Total sample volume [mL]	HPLC gradient
**1a**	40	Initial: 3% ACN, 97% water<br>10 min: 3% ACN, 97% water<br>18 min: 40% ACN, 60% water<br>18.5 min: 3% ACN, 97% water<br>20 min: 3% ACN, 97% water
**1b**	30	
**2a**	40	Initial: 3% ACN, 97% water<br>7 min: 3% ACN, 97% water<br>18 min: 40% ACN, 60% water<br>18.5 min: 3% ACN, 97% water<br>20 min: 3% ACN, 97% water
**2b**	10	

A flow rate of 21.24 mL min^{-1} was used. Acetonitrile (ACN) and deionized water were applied as solvents. Samples were analysed at 210 nm.

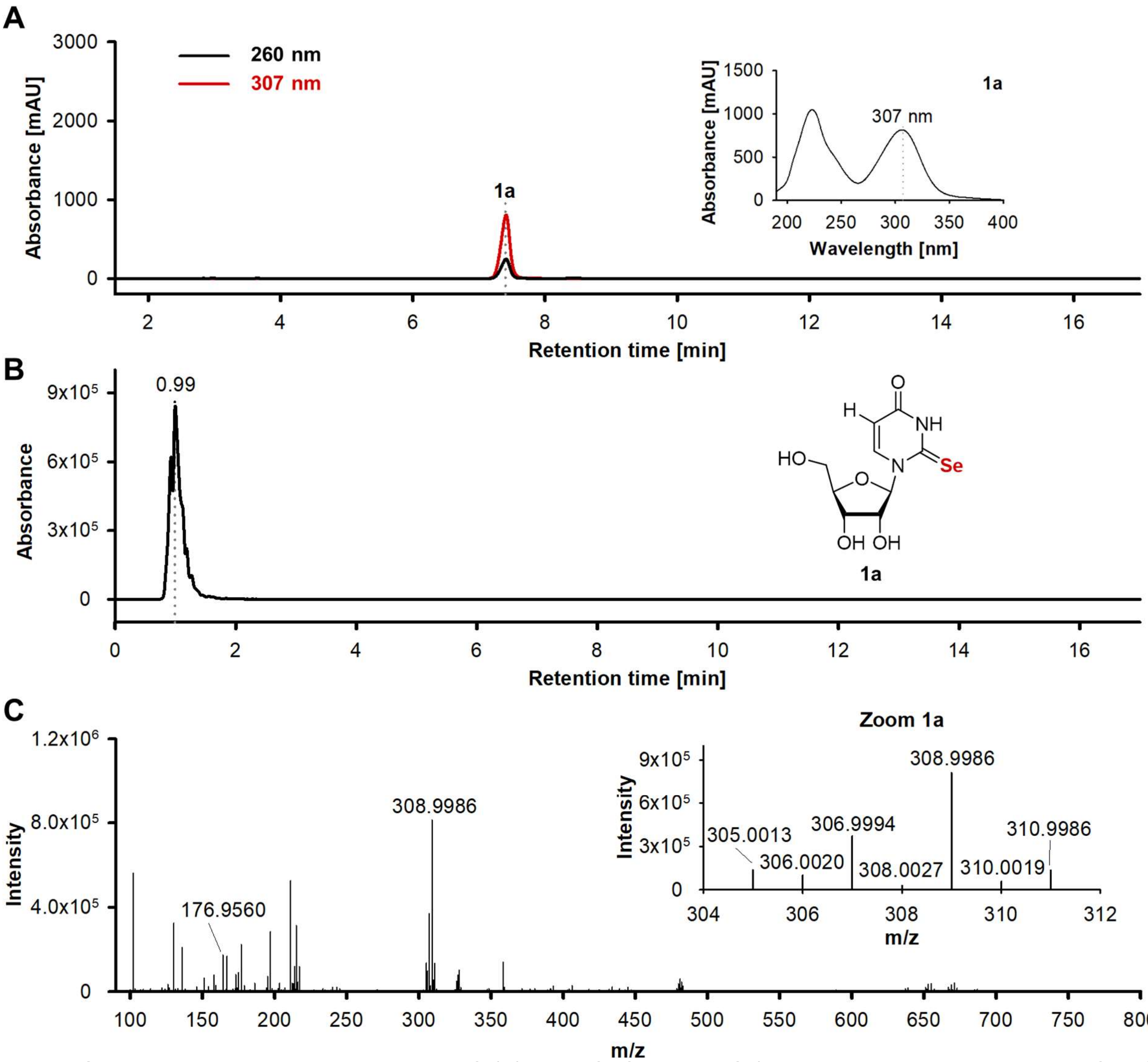

Figure S6. Product **1a** was analysed by HPLC (A) and ESI-Orbitrap-MS (B: extracted ion chromatogram, C: MS spectrum).

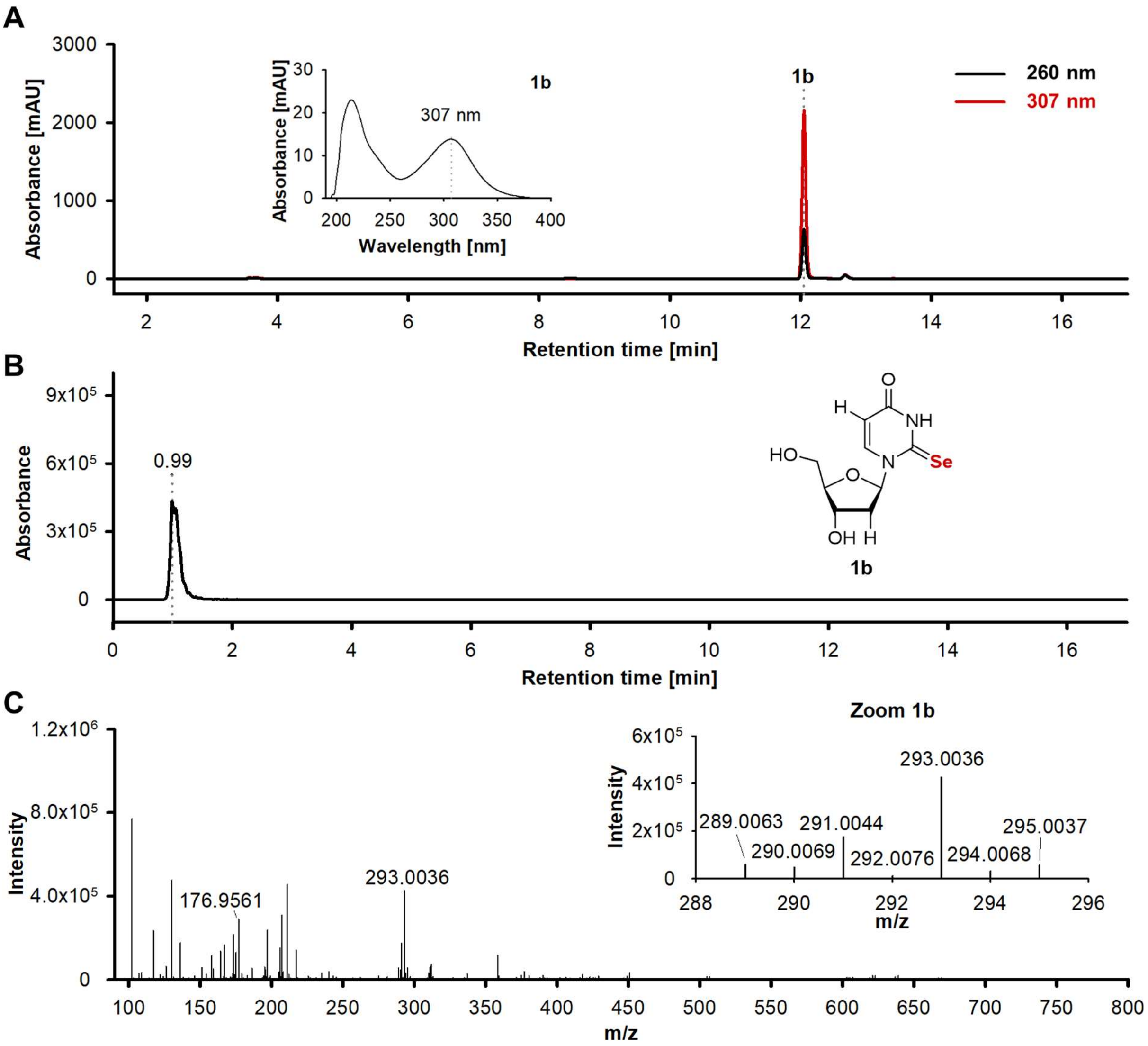

Figure S7. Product **1b** was analysed by HPLC (A) and ESI-Orbitrap-MS (B: extracted ion chromatogram, C: MS spectrum).

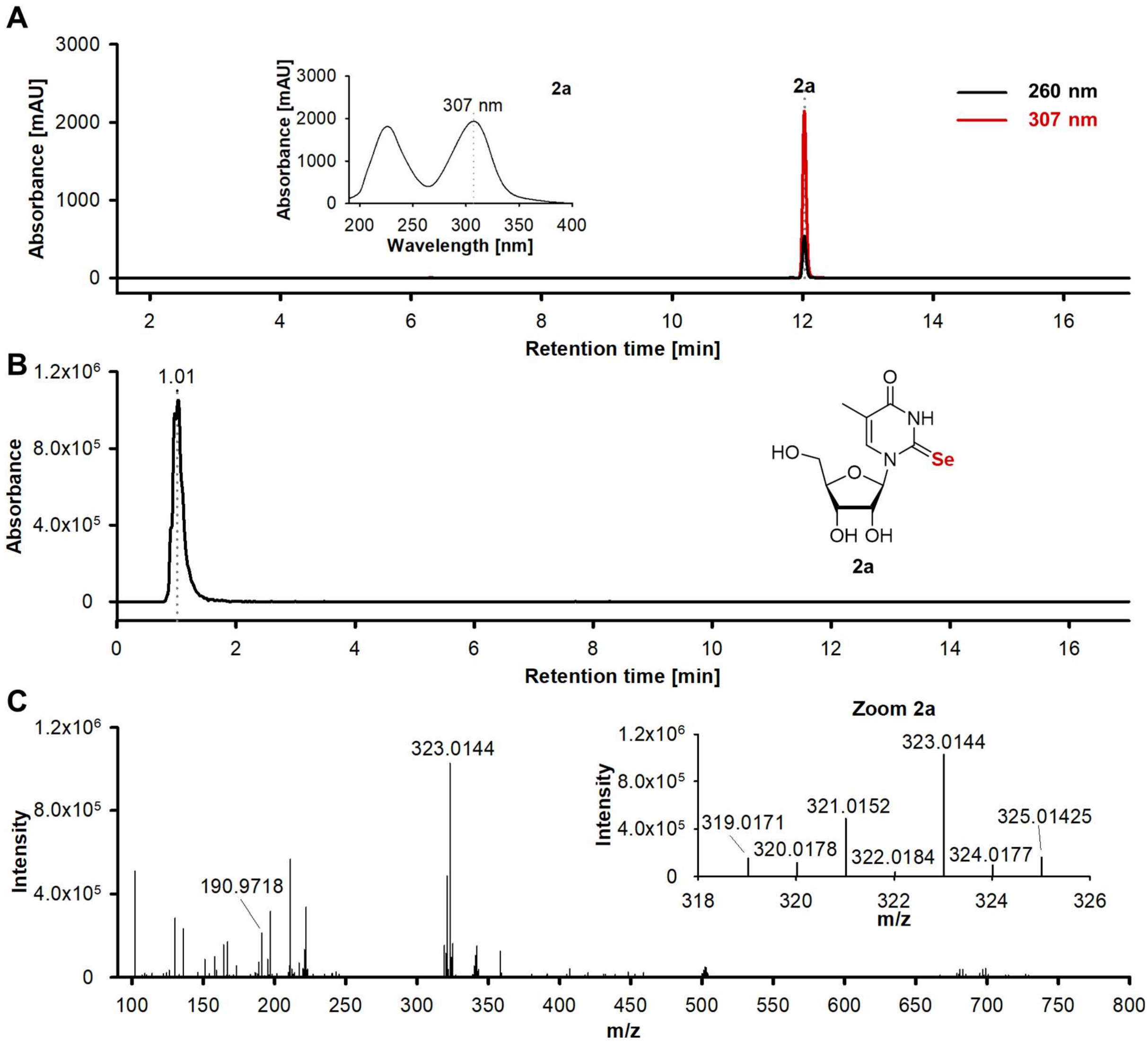

Figure S8. Product **2a** was analysed by HPLC (A) and ESI-Orbitrap-MS (B: extracted ion chromatogram, C: MS spectrum).

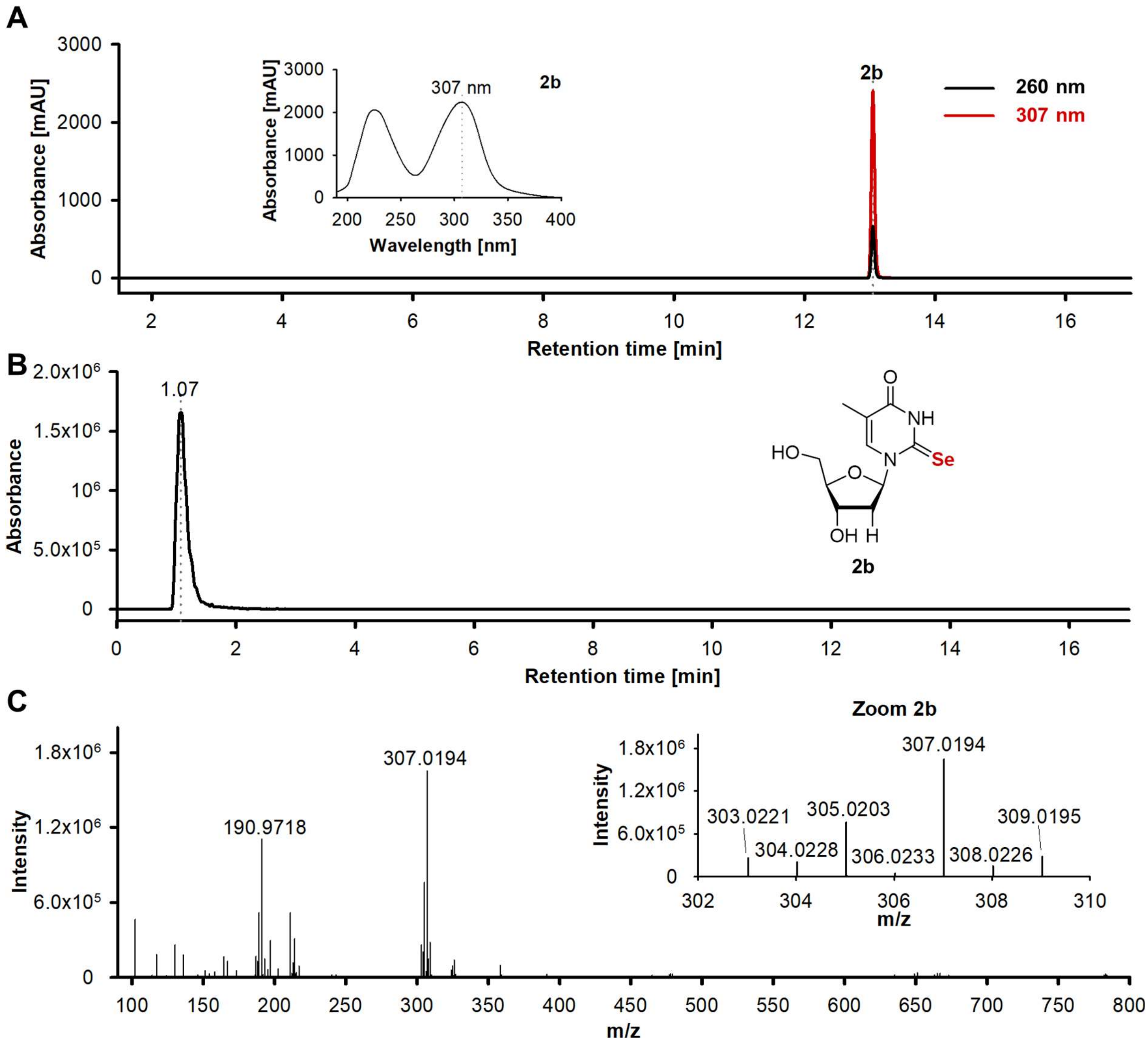

Figure S9. Product **2b** was analysed by HPLC (A) and ESI-Orbitrap-MS (B: extracted ion chromatogram, C: MS spectrum).

References

[1] A. Brand, L. Allen, M. Altman, M. Hlava, J. Scott, *Learn. Publ.* **2015**, *28*, 151–155.

[2] F. Kaspar, K. F. Hellendahl, *Zenodo* **2020**, DOI 10.5281/zenodo.4302012.

[3] F. Kaspar, R. T. Giessmann, S. Westarp, K. F. Hellendahl, N. Krausch, I. Thiele, M. C. Walczak, P. Neubauer, A. Wagner, *Chembiochem* **2020**, DOI 10.1002/cbic.202000204.

[4] F. Kaspar, R. T. Giessmann, P. Neubauer, A. Wagner, M. Gimpel, *Adv. Synth. Catal.* **2020**, *362*, 867–876.

[5] F. Kaspar, *Zenodo* **2020**, DOI 10.5281/ZENODO.3723806.
